# Mussel-Inspired Dopamine and Carbon Nanotube Leading to a Biocompatible Self-Rolling Conductive Hydrogel Film

**DOI:** 10.3390/ma10080964

**Published:** 2017-08-18

**Authors:** Junzi Jiang, Yong Huang, Yitian Wang, Hui Xu, Malcolm Xing, Wen Zhong

**Affiliations:** 1 Department of Mechanical Engineering, University of Manitoba, Winnipeg, MB R3T 2N2, Canada; xiaobeike000@163.com; 2 Department of Biosystem Engineering, University of Manitoba, Winnipeg, MB R3T 2N2, Canada; wangy392@myumanitoba.ca (Y.W.); umxu68@myumanitoba.ca (H.X.); 3 Chongqing Academy of Animal Sciences, Chongqing 402460, China; huangyongcqbb@126.com

**Keywords:** mussel-inspired dopamine, self-rolling film, self-assembly, carbon nanotube conductive film, cellular compatibility

## Abstract

We report a novel self-rolling, conductive, and biocompatible multiwall carbon nanotube (MWCNT)-dopamine-polyethylene glycol (PEG) hydrogel film. The gel can self-fold into a thin tube when it is transferred from a glass slide to an aqueous environment, regardless of the concentrations of the MWCNT. The film presents a highly organized pattern, which results from the self-assembly of hydrophilic dopamine and hydrophobic carbon nanotubes. By exploring the biomedical potential, we found that MWCNT-included rolled film is nontoxic and can promote cell growth. For further functional verification by qPCR (quantitative polymerase chain reaction), bone marrow derived mesenchymal cells present higher levels of osteogenic differentiations in response to a higher concentration of CNTs. The results suggest that the self-rolling, conductive CNT-dopamine-PEG hydrogel could have multiple potentials, including biomedical usage and as a conductive biosensor.

## 1. Introduction

A most efficient and popular method for tissue regeneration involves a three dimensional (3D) matrix that provides a stable and tissue-mimicking environment for cell proliferation and differentiation [1,2,3,4,5]. An ideal matrix or cell living environment is generally expected to have an appropriate morphology, mechanical properties, and biological compatibility. It has been a challenge to fabricate an artificial 3D matrix that meets all these requirements. Both natural and synthetic materials have been used to construct 3D matrixes for tissue engineering. However, natural materials are generally known to have poor mechanical properties, while synthetic materials often lack cell recognizable receptors. As a result, a combination of these two types of materials may be a promising solution for tissue regeneration.

Marine mussels are known for their exceptional adhesion performance; they attach to various surfaces in a humid environment. Much evidence demonstrates that the repeated 3,4-dihydroxy-l-phenylalanine-lysine (DOPA-K) motif has a main contribution to mussels’ adhesion properties [6]. Lee et al. cross-linked poly(ethylene glycol) diacrylate with N-methacrylated DOPA to form hydrogels, which have appreciable elastic moduli for tissue regeneration applications [7]. Dopamine molecules have a similar structure and adhesive capacity to DOPA [8]. Several studies also indicate that dopamine can adhere to cells [9,10,11,12,13,14,15,16]. Dopamine was coated onto the super-hydrophobic surface of poly(tetrafluoroethylene) to improve its cell adhesion [17]. In vitro tests showed that cell adhesion and proliferation on the dopamine-modified poly(tetrafluoroethylene) were significantly higher than that of the control group. Dopamine was also coated onto a patterned silicon wafer to guide the growth of three types of cells [18]. All cells were found to have a better affinity to the dopamine-coated areas. All these works have demonstrated that dopamine is a promising material for biomedical applications.

Since Iijima’s landmark paper was published in 1991, carbon nanotubes (CNTs) have been attracting extensive research efforts because of their outstanding properties [19]. CNTs have large surface areas, preferable electrical properties, nanogeometry, low density, and good tissue compatibility, making them suitable for application such as biomedicine and tissue regeneration [20,21,22,23]. The unique structure of CNTs, a hollow cylindrical carbon tube, provides a framework onto which functional molecules or polymers can be incorporated that is critical for biomedical applications [24,25]. CNTs have also been used in the development of carbon nanotube hybrid hydrogels for biomedical applications [26,27]. CNTs can be non-covalently coated by dopamine molecules via π-π stacking between the CNTs and the benzene rings of dopamine [28,29]. The enhanced dispersion of CNTs in aqueous environments was also observed after being modified by dopamine due to dopamine hydrophilicity [30].

There are generally two approaches to fabricate tissue engineering scaffolds: ‘top-down’ [31,32] and ‘bottom-up’ [33,34,35]. For most top-down scaffolds, cells have difficulty spreading into the scaffolds homogenously. Bottom-up scaffolds, on the other hand, allow cells to distribute more evenly in the scaffolds but often trap the cells and limit their activities. A novel alternative approach is to wrap cells into tissue engineering scaffolds using self-rolling tubes [36,37,38]. Cell-embedded tubes can self-assemble to form a complex microporous 3D structure [39]. Self-rolling tubes are similar to osseous and nervous tissues in shape [40] and therefore are promising for applications in biomedicine [41,42,43]. Luchnikov et al. developed a polymer-based bilayer film made of poly(4-vinylpyridine) and polystyrene. Such films self-fold under acidic conditions [44]. Kalaitzidou et al. [45,46] designed a thermo-controlled self-rolling Polydimethylsiloxane (PDMS)/gold film. Gracia et al. demonstrated that a patterned SU-8 photoresist-polycaprolactone film rolls at 60 °C [36]. Stoychev et al. also reported a thermo-sensitive self-folding polycaprolactone/poly(N-isopropylacrulamide) bilayer film, which is responsive to temperature changes (25 °C to 33 °C) to capture or release cells [47]. The clinical applications of these self-folding materials, however, have been limited by such issues as poor biocompatibility and the requirement of an external stimulus.

In this study, we designed a facile photo-initiated self-rolling film by a combination of dopamine crosslinker, multiwall carbon nanotubes (MWCNTs), and poly(ethylene glycol) diacrylate (PEGDA). PEGDA was chosen as the backbone polymer to form the gel because of its biocompatibility and appropriate mechanical properties for a scaffold. The crosslinker was synthesized though Michael-addition of dopamine and N′N′-methylene-bis-acrylamide. Such an approach introduces double bonds to dopamine and further crosslinks dopamine to the backbone of the structure. This study shows that the self-folding film has good conductivity, biocompatibility, cell attachment, and bio-effect on cell differentiation.

## 2. Materials and Method

### 2.1. Materials

Poly(ethylene glycol) diacrylate (PEGDA, *Mw* = 700), F-127, dopamine, and N′N′-methylene-bis-acrylamide (MBA) were all ordered from Sigma-Aldrich (Oakville, ON, Canada). Igacure@2959 was ordered from BASF Canada (Mississauga, ON, Canada). All chemicals were used without further purification. Phosphate buffered saline (PBS) 1 × powder, Hoechst 33342, BODIPY FL phallacidin, To-Pro-3 iodide, and a Live-Cell staining kit were all purchased from Invitrogen (Carlsbad, CA, USA). 3-(4,5-dimethyl thiazolyl-2)-2,5-diphenyltetrazolium bromide (MTT) cell viability assay kits were from Biotium Inc. (Hayward, CA, USA). Optimal cutting temperature (OCT) compounds were purchased from VWR Canada (Mississauga, ON, Canada). Pristine multi-walled carbon nanotubes (MWCNTs, 95% purity) were purchased from Timesnano (Chengdu, China) and used without further purification. The morphology of the MWCNT was examined under a FEI Talos F200X Transmission electron microscopy (TEM, Hillsboro, OR, USA).

### 2.2. Synthesis of Dopamine-MBA Crosslinker

Deionized water/ethanol (*v/v* = 4:3) solution was adjusted to pH 6 by using 0.5 M hydrochloric acid in order to protect dopamine from oxidization. 500 mg of MBA (3 mmol) was then dissolved into the above solution to achieve a concentration of 70.1 mg/mL. After that, 475 mg of dopamine (2 mmol) was added to the solution under nitrogen protection to exclude oxygen. The reaction was conducted in darkness in an oil bath at 45 °C and under constant stirring for three days. The dopamine-MBA crosslinker was obtained after lyophilization and stored at −20 °C.

### 2.3. Preparation of MWCNT-Dopamine-PEG Hydrogels

24 mg MWCNTs were dispersed in 10 (*v/w*)% F-127 solution homogeneously in a ultrasonic mixer for 1 h. 38 and 80 µL of MWCNT solutions, respectively, were mixed with 200 µL of 2 (*v/w*)% crosslinker solution in the ultrasonic mixer for 30 min and were then blended with 1 mL 20 (*v/w*)% PEGDA and 130 µL 10 (*v/w*)% Igacure 2959 to form a precursor solution. 100 µL precursor solution was dropped onto a 25 mm × 25 mm glass slip and covered with another glass slip of the same size. The solution embedded between the slips was put under a UV light for 15 min to form a hydrogel. Control samples of hydrogels without MWCNT were also prepared using the same method described above.

### 2.4. Fourier Transform Infrared (FTIR) Spectroscopy

FTIR was used to characterize the prepared hydrogels. The prepared hydrogels were freeze-dried for two days. Then the dried hydrogel samples were ground to powder, mixed with KBr powder, and compressed into pellets. FTIR spectra were recorded on a Thermo Scientific Nicolet Is10 FT-IR Spectrometer (Ottawa, ON, Canada).

### 2.5. Cell Culture

Mouse bone marrow stromal cell lines (BMSCs from ATCC, USA) were cultured with Dulbecco’s Modified Eagle’s Medium (DMEM, Thermo Fisher, Ottawa, ON, Canada) supplemented with 10% fetal bovine serum (FBS, Thermo Fisher, Ottawa, ON, Canada), 1.0 × 10^5^ U1-1 penicillin (Sigma, Oakville, ON, Canada) and 100 mg/L streptomycin (Sigma, Oakville, ON, Canada) at 37 °C in 5% CO_2_.

### 2.6. Cell Growth on Hydrogels

All the hydrogels with or without MWCNTs were immersed in PBS (pH = 7.4), which was changed every three hours. After 36 h, the hydrogels were taken out from the PBS and immersed in cell culture medium for another 12 h. Then the hydrogels were placed in cell culture dishes (35 mm × 10 mm), which were pre-coated with agarose gel to avoid cell adhesion. Cells were seeded onto the hydrogels and incubated for growth.

### 2.7. Cell Viability

BMSCs were used for evaluating cell viability in MTT assay. All the hydrogels were cut by a punch into circular disks of radius 6 mm, which perfectly fit in the wells in a 96-well plate. 1 × 10^4^ BMSCs were cultured in the hydrogel in each well of a 96-well culture plate and the media was changed every two days. At certain time intervals, 10 µL of MTT reagent was mixed to each well followed by 4 h incubation. After the MTT solution was removed, 200 µL of DMSO was added to each well to dissolve the crystals. The absorbance was measured at 595 nm (*n* = 3) on a microplate reader. Live/Dead staining was used for evaluating the cytotoxicity of the hydrogels. The hydrogels were prepared between two cover slips, according to the method described before. BMSC were cultured on the hydrogels and dyed at certain time intervals. Cells with hydrogels were immersed in PBS mixed with 2 mM calcein AM (acetoxymethyl) and 4 mM ethidium homodimer probes for 20 min. After that, the hydrogels with cells were rinsed with PBS and imaged under a florescence microscope.

### 2.8. Cell Morphology

The morphology of BMSC-laden hydrogels was observed under a florescence microscope. The cells on hydrogels were immersed in 4% paraformaldehyde solution at room temperature for 30 min. After being washed with PBS, the samples were permeabilized using 0.5% Triton X-100 in PBS solution at room temperature for 5 min. They were then blocked in 1% bovine serum albumin PBS solution at room temperature for 10 min. The samples were incubated in FITC (Fluorescein isothiocyanate) solution for 20 min at room temperature and stained with TO-PRO3 (a nucleic acid-binding dye that stains early apoptotic and necrotic cells differentially) before being examined under a confocal Laser scanning microscopy (CLSM).

### 2.9. Electrical Conductivity Test

All the electrical conductivity tests were conducted on a Multifunction Digital Four-probe Tester (Suzhou Jingge Electronic Co. Ltd, Suzhou, China).

### 2.10. Quantitative Real-Time PCR Analysis

100 mL BMSCs solution with a concentration of 2 × 10^6^ cell/mL was seeded into the hydrogel. After two days incubation, the samples were moved into DMEM mixed with 50 mg/mL L-ascorbic acid 1-phosphate, 10 mM b-glycerophosphate, and 100 nM dexamethasone. The medium was refreshed every two days. At a certain time point, the medium was carefully moved out without damaging cells and was rinsed with DPBS for three times. Then the samples with cells were frozen by liquid nitrogen and grounded into small pieces. The total RNA isolation and cDNA sythesis were performed to analyze the osteogenic differentiation based on standard procedures. SYBER Green assays were used to conduct quantitative real-time PCR (quantitative polymerase chain reaction).

In this study, we used the control group as a standard sample. For example, the formula of ALP (alkaline phosphatase) gene expression of 0.7 mg/mL WCNT-dopamine-PEG is as follows:

$$\mathrm{ALP}\;\mathrm{gene}\;\mathrm{expression}=2^{-\left[\left(C_{\mathrm{target}}-C_{\mathrm{ALP}}\right)-\left(C_{\mathrm{target}-0}-C_{\mathrm{ALP}-0}\right)\right]}$$

in which $C_{\mathrm{target}\;}$ is the GAPDH gene expression of 0.5 wcnt-gel, $C_{\mathrm{ALP}}$. is the ALP gene expression of 0.7 mg/mL WCNT-dopamine-PEG, $C_{\mathrm{target}-0}$. is the GAPDH gene expression of the control group, and $C_{\mathrm{ALP}-0}$ is the ALP gene expression of the control group.

## 3. Result and Discussion

### 3.1. Characterization of Self-Folding Film

**Figure 1:**
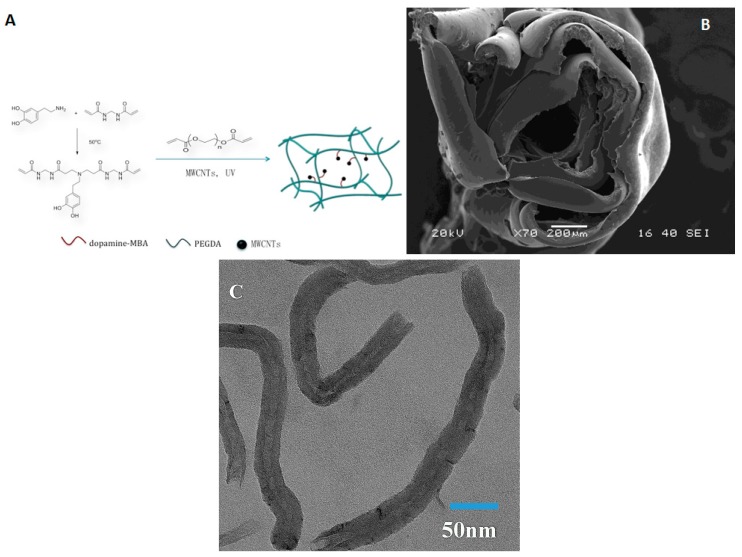
(**A**) Schematic of fabrication of the dopamine crosslinker and self-rolling film; (**B**) Scanning electron microscopic (SEM) image of multiwall carbon nanotube (MWCNT)-dopamine- polyethylene glycol (PEG) film’s self-rolling (Video S1 in Supplementary); (**C**) Transmission electron microscopic (TEM) image of MWCNTs.

**Figure 2:**
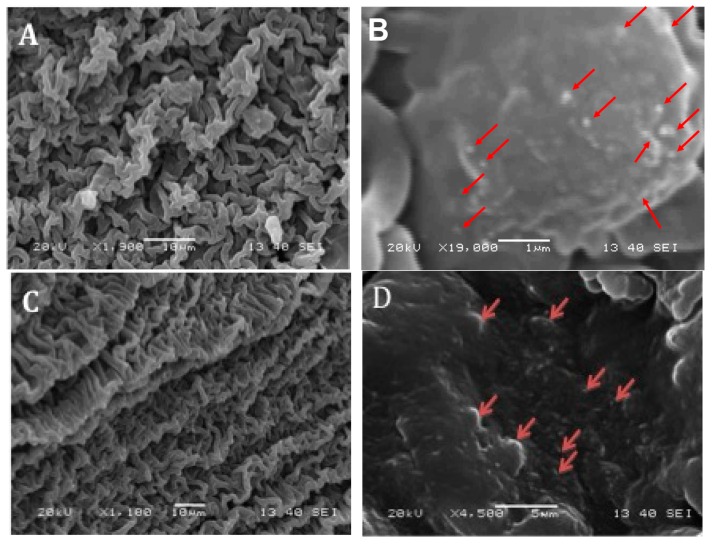

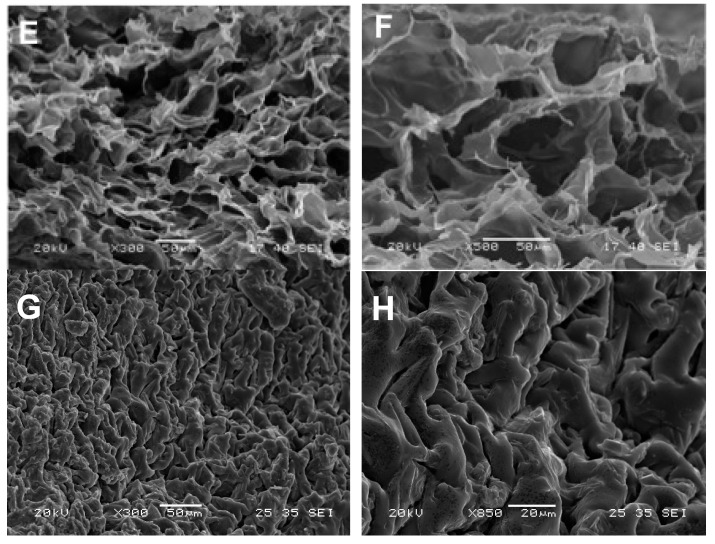
SEM images of (**A**) 0.7 mg/mL MWCNT-dopamine-PEG film surface; (**B**) high-resolution 0.7 mg/mL MWCNT-dopamine-PEG film surface; (**C**) 1.4 mg/mL MWCNT-dopamine-PEG film surface; (**D**) high-resolution 1.4 mg/mL MWCNT-dopamine-PEG film surface (MWCNTs are pointed out by red arrows); (**E**) PEG film surface; (**F**) high-resolution PEG film surface; (**G**) dopamine-PEG film surface; (**H**) high-resolution dopamine-PEG film surface.

TEM images of MWCNTs were shown in Figure 1C. The MWCNTs have an average diameter of 35 nm and an average length of 400 nm. The self-rolling film was synthesized from the polymerization between PEG and the dopamine crosslinker under UV light (Figure 1A). The thin film was formed between two glass slides and can be torn off from the slides. The film automatically formed into a roll in less than 30 s after being put into water (Figure 1B, movie 1). A scanning electron microscope (SEM) was used to examine the surface morphology of the hydrogel films (Figure 2). Interestingly, as can be seen in the SEM images, regardless of the concentrations of MWCNTs, the surfaces of the hydrogels are consistently wrinkled (Figure 2A,C). However this wrinkled structure was not observed in the SEM image of the PEG gel, and the PEG-Dopamine gel and the dopamine introduced a self-resemblance between hydrophilic PEG and hydrophobic MWCNTs (Figure 2F–H). In the high-resolution images, MWCNTs are indicated by red arrows and are shown to be embedded in the films. Apparently more MWCNTs can be found in the 1.4 mg/mL MWCNT film than in the 0.7 mg/mL MWCNT film (Figure 2B,D).

The underling mechanism may result from the interactions between hydrophobic CNTs and hydrophobic polydopamine crosslinked membranes. The self-rolled membrane can be used as a neural guide tube for neutral regeneration, and the membrane can also be applied to cardiovascular reconstruction due to its biocompatible and conductive properties. Additionally, the self-assembled pattern that appeared is good as a model to verify the hydrophobic component’s role and contribution in making the varied interactions with hydrophilic matrix by tuning the concentrations of hydrophobic CNTs.

**Figure 3:**
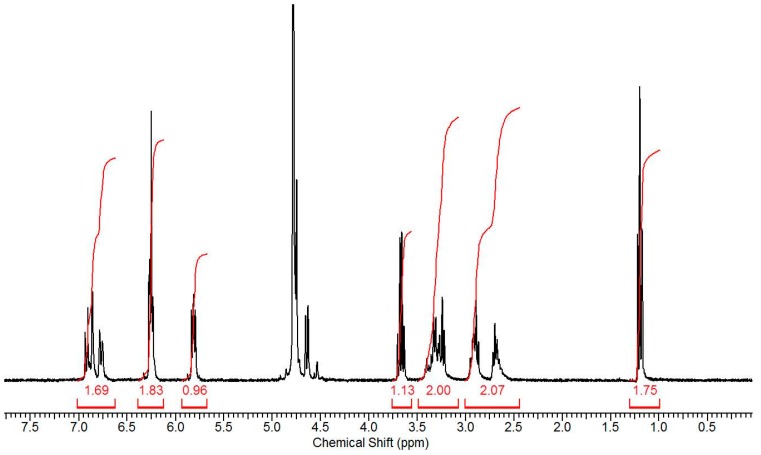
^1^H NMR spectra of the crosslinker (dopamine-N′N′-methylene-bis-acrylamide (MBA)).

The chemical structure of the dopamine-MBA crosslinker was confirmed by ^1^H NMR spectra (Figure 3). The integrals of peaks at 5.7 and 6.2 ppm represent the residual vinyl bonds in the polymers [48]. Furthermore, the integral of peak at 6.8 ppm indicates the extent of the benzene ring. The peaks at 3.3 and 2.8 ppm are ethyl groups from dopamine. The peaks at 4.6 ppm are ethyl groups from MBA. The peaks at 2.4 are N-H bond from dopame and MBA. The NMR spectra showed that the dopamine has been successfully linked with the MBA. The vinyl residual as an end group was involved in radical polymerization and served as a macromolecular crosslinker.

### 3.2. Live/Dead Assay

**Figure 4:**
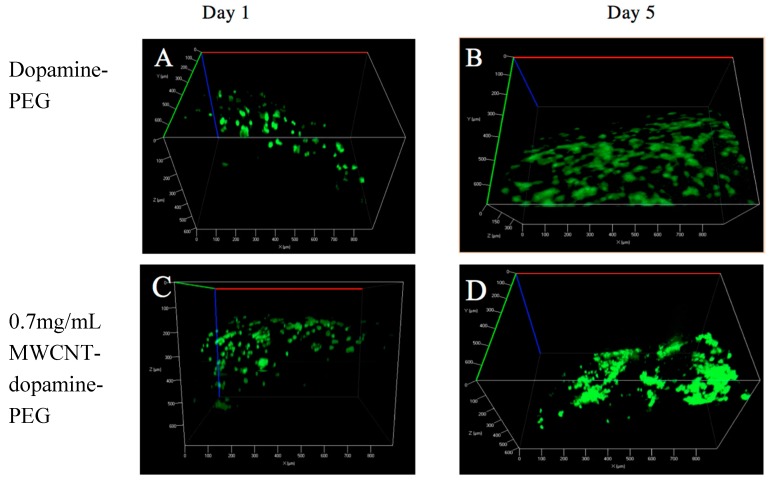

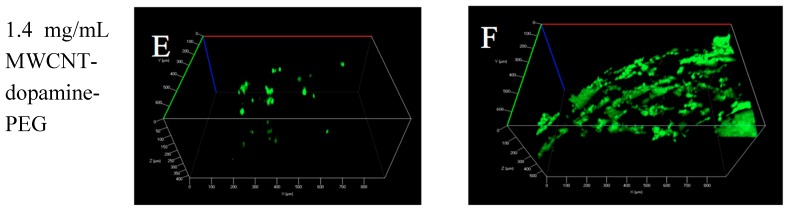
Live/dead test of bone marrow stromal cells (BMSCs) cultured on dopamine-PEG hydrogel on (**A**) day 1 and (**B**) day 5; on 0.7 mg/mL MWCNT-dopamine-PEG hydrogel (**C**) day 1 and (**D**) day 5; on 1.4 mg/mL MWCNT-dopamine-PEG hydrogel (**E**) day 1 and (**F**) day 5. (Live cells are green and dead cells are red).

Live/dead staining was employed to assess cell viability. BMSCs were cultured on the dopamine-PEG hydrogels with or without MWCNT of different concentrations (0.7 mg/mL and 1.4 mg/mL respectively). As shown in Figure 4, after one day incubation, most BMSCs (almost 100%) were found to be attached to all three kinds of hydrogels and alive. After five days culture, the number of BMSCs as well as the degrees of cell spreading significantly increased on all samples, with or without MWCNT. The results suggest that MWCNTs barely have any influence on the biocompatibility or cell adhesion of dopamine hydrogels. Furthermore, through a fluorescence microscope, it was clearly observed that cells were spread all over the curves, demonstrating that the rolling shape did not have any negative effect on the viability of the cells.

### 3.3. MTT Assay

**Figure 5:**
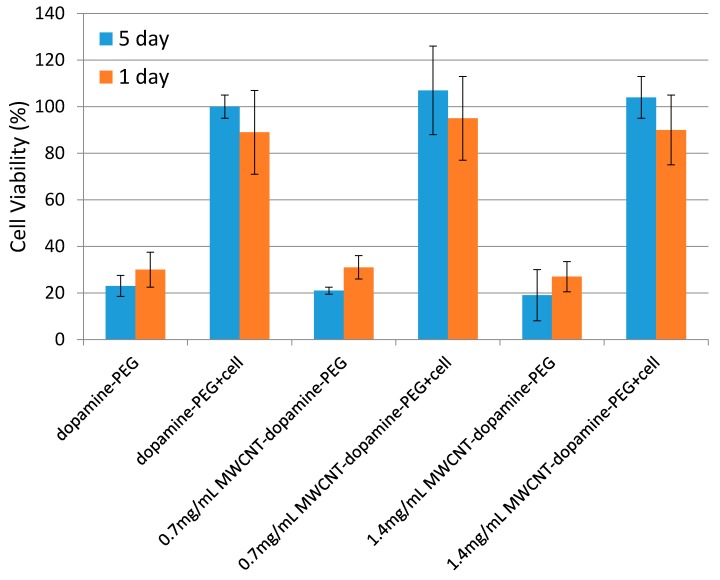
MTT assay of BMSCs cultured on dopamine-PEG, 0.7 mg/mL MWCNT-dopamine-PEG, and 1.4 mg/mL MWCNT-dopamine-PEG gels on day 1 and day 5.

Mouse bone marrow stromal cells (BMSCs) were seeded onto the hydrogels. An MTT test was used to assess the cell viability for day 1 and day 5. The viability of BMSCs cultivated on tissue culture plates (TCPS) was considered as the standard of 100%. Hydrogels without MWCNTs were also tested as a control. As shown in Figure 5, hydrogels with MWCNTs had similar cell viability to hydrogels without MWCNT after culturing for 1 day and 5 days, confirming the live/dead assay results that MWCNTs are harmless to the cells. After 5 days of incubation, the cell viability of all the samples, with and without MWCNTs, significantly increased, and there was no significant difference between hydrogels with and without MWCNT.

### 3.4. Cell Morphology

**Figure 6:**
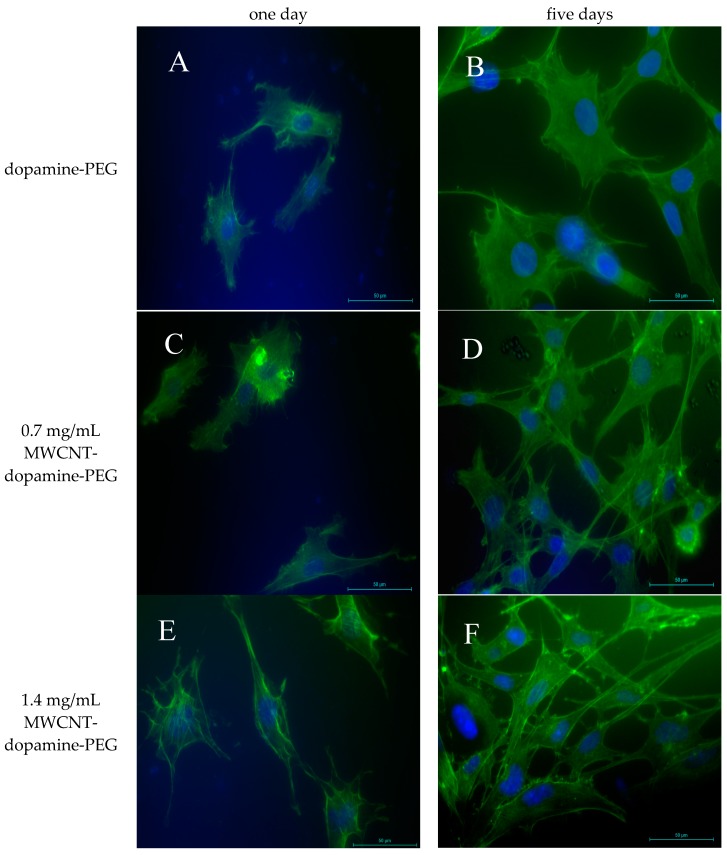
BMSCs cell morphology on dopamine-PEG on (**A**) day 1 and (**B**) day 5; on 0.7 mg/mL MWCNT-dopamine-PEG on (**C**) day 1 and (**D**) day 5; and on 1.4 mg/mL MWCNT-dopamine-PEG hydrogels on (**E**) day 1 and (**F**) day 5. The scale bars for all images are 20 μm. Green stands for cytoskeleton and blue stands for nucleus for all images.

The cell morphology of BMSCs cultured on the hydrogels was also studied by a fluorescence microscope. BMSCs were cultured on hydrogels with different concentrations of MWCNTs to examine the effect of MWCNT concentration on the cell morphology. As shown in Figure 6, after one day, cells were observed to be attached to all samples, which is consistent with the live/dead assay. However, after five days, the cytoskeletons of BMSCs on hydrogels with MWCNT seemed to have longer and more organized morphologies compared to cells seeded on the hydrogels without MWCNTs. The higher the concentration of MWCNTs, the better the spreading out morphology, indicating that MWCNTs may have some influence on cell morphology.

### 3.5. Conductivity

**Figure 7:**
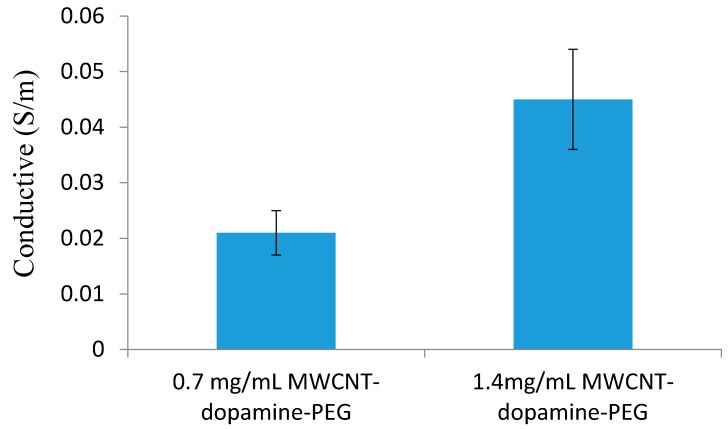
Electric conductivity of 0.7 mg/mL and 1.4 mg/mL of MWCNT-dopamine-PEG gels.

The electrical conductivity of MWCNT-dopamine-PEG hydrogels was measured using the standard four-probe method. In order to obtain accurate results, we prepared three hydrogels with different MWCNT concentrations and measured the electrical conductivity at 30 spots on each sample. As shown in Figure 7, hydrogels without MWCNTs have a conductivity of 0 s/m at room temperature. However, the electrical conductivity of 0.7 mg/mL MWCNT hydrogels increases to 0.02 s/m, which is almost half of the electrical conductivity of the 1.4 mg/mL WCNT hydrogels. This indicates that the MWCNT hydrogels have good conductivity and that their conductivity can be tuned by varying the concentration of MWCNT.

### 3.6. RTPCR

**Figure 8:**
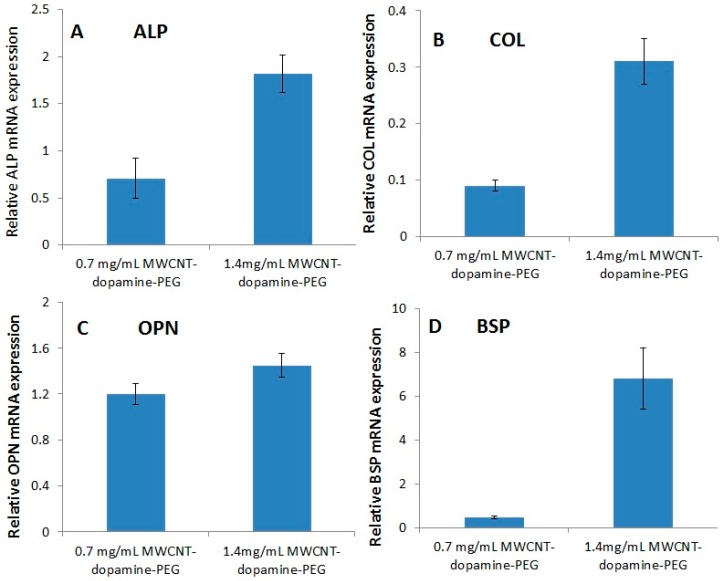
Real time qPCR of osteogenic gene expression levels of BMSCs cultured in vitro. Total RNA was prepared from BMSCs grown on hydrogels for seven days. (**A**) ALP (alkaline phosphatase); (**B**) COL (collagen I); (**C**) OPN (Osteopontin) and (**D**) BSP (bone sialoprotein) gene expressions were quantified using real time-qPCR methods; GAPDH was used as an internal control. Data values are expressed as mean ± SE (*n* = 3)

Bone sialoprotein (BSP) is one of the most curial proteins for bone differentiation. Its expression can be found in scleroblastsor. On day 2, the BSP genic expression was slightly increased. On day 7, the rate of growth became even higher (Figure 8B). Collagen (COL) is a critical marker in the late stage of bone differentiation [49]. The expression level of COL (Figure 8D) had the similar tendency to that of BSP. Osteopontin (OPN) is another important human gene product, which is related to osteogenesis [49]. It plays a role in the organic linking component of bone. As shown in Figure 8C, the expression profiling of OPN was relatively lower than that of both BSP and COL, but the expression profiling of OPN still have a positive trend after seven days during the osteogenetic differentiation. Alkaline phosphatase (ALP) is an enzyme that exists in all tissues in human body, which can remove the phosphate group from nucleotides or proteins. ALP may be a marker for bone metabolism: a high level of ALP can be observed when active bone is formed [50]. The ALP expression was evaluated when the BMSCs were cultured on hydrogels. The results show that the hydrogels had a high ALP expression level throughout one week (Figure 8A).

## 4. Conclusions

We developed a novel, self-rolling, conductive, and biocompatible multiwall carbon nanotube (MWCNT)-dopamine-PEG hydrogel film for tissue engineering. This film can self-fold as it is transferred from the air to the water regardless of the concentration of MWCNTs. In vitro tests proved that MWCNT is nontoxic and promotes cell growth. The real time-qPCR test indicated that cells have higher level of differentiation with higher concentrations of MWCNTs. This suggests that the self-rolling and conductive MWCNT-dopamine-PEG hydrogel is a promising scaffolding material for bone regeneration.

## Supplementary Materials

The following are available online at www.mdpi.com/1996-1944/10/8/964/s1, Video S1: Self-rolling of multiwall carbon nanotube (MWCNT)-dopamine- polyethylene glycol (PEG) film.
